# Modeling a Novel Variant of Glycogenosis IXa Using a Clonal Inducible Reprogramming System to Generate “Diseased” Hepatocytes for Accurate Diagnosis

**DOI:** 10.3390/jpm12071111

**Published:** 2022-07-07

**Authors:** Guillem Garcia-Llorens, Sergi Lopez-Navarro, Teresa Jaijo, Jose V. Castell, Roque Bort

**Affiliations:** 1 Unidad de Hepatología Experimental y Trasplante Hepático, Instituto de Investigación Sanitaria La Fe, Hospital Universitario y Politecnico La Fe, 46026 Valencia, Spain; guillem_garcia@iislafe.es (G.G.-L.); serlona@alumni.uv.es (S.L.-N.); jose.castell@uv.es (J.V.C.); 2 Biochemistry and Molecular Biology Department, Universidad de Valencia, 46026 Valencia, Spain; 3 Centro de Investigación Biomédica en Red de Enfermedades Hepáticas y Digestivas (CIBERehd), Instituto de Salud Carlos III, 28029 Madrid, Spain; 4 Molecular, Cellular and Genomic Biomedicine, Instituto de Investigación Sanitaria La Fe, Hospital Universitario y Politecnico La Fe, 46026 Valencia, Spain; jaijo_ter@gva.es

**Keywords:** glycogen, GSD type IX, hepatocyte-like cells, direct reprogramming, high throughput

## Abstract

The diagnosis of inherited metabolic disorders is a long and tedious process. The matching of clinical data with a genomic variant in a specific metabolic pathway is an essential step, but the link between a genome and the clinical data is normally difficult, primarily for new missense variants or alterations in intron sequences. Notwithstanding, elucidation of the pathogenicity of a specific variant might be critical for an accurate diagnosis. In this study, we described a novel intronic variant c.2597 + 5G > T in the donor splice sequence of the PHKA2 gene. To investigate PHKA2 mRNA splicing, as well as the functional consequences on glycogen metabolism, we generated hepatocyte-like cells from a proband’s fibroblasts by direct reprogramming. We demonstrated an aberrant splicing of PHKA2, resulting in the incorporation of a 27 bp upstream of intron 23 into exon 23, which leads to an immediate premature STOP codon. The truncated protein was unable to phosphorylate the PYGL protein, causing a 4-fold increase in the accumulation of glycogen in hepatocyte-like cells. Collectively, the generation of personalized hepatocyte-like cells enabled an unequivocal molecular diagnosis and qualified the sister’s proband, a carrier of the same mutation, as a candidate for a preimplantation genetic diagnosis. Additionally, our direct reprogramming strategy allows for an unlimited source of “diseased” hepatocyte-like cells compatible with high-throughput platforms.

## 1. Introduction

Inherited metabolic disorders (IMDs) are a heterogeneous group of genetic pathologies, frequently with a complicated prognosis due to the affection of important biochemical pathways and essential processes for the body’s metabolism. The genetic defects of the IMDs lead to structural or functional alterations of proteins that produce a deregulation in the human metabolism, generating a clinical manifestation of the disease. Recent data, considering the increasing number of new disorders and their underestimation, indicate that the incidence of IMDs is 1 in 800 live births [1]. Around 700 IMDs have been identified, which are grouped into alterations in amino acid metabolism, urea cycle, fatty acid beta-oxidation, organic acid metabolism, carbohydrate metabolism, lysosomal storage, purine and pyrimidine metabolism, blood disorders, mitochondrial disorders, peroxisomal diseases, and others [1].

**Figure 1:**
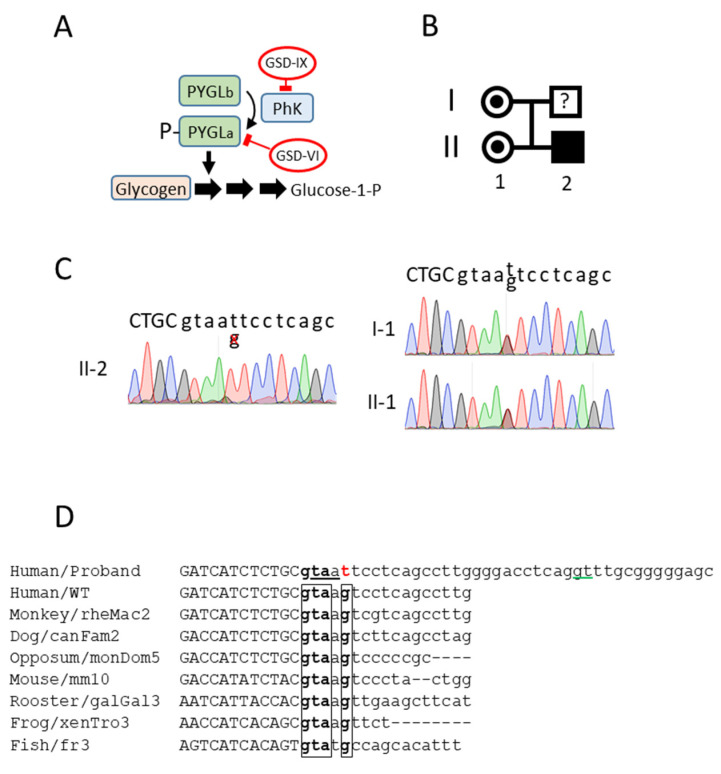
PHKA2 novel mutation. (**A**) The role of glycogen phosphorylase (PYGL) and phosphorylase kinase (PhK) in glycogen catabolism. (**B**) Family tree of the proband. (**C**) Sanger sequencing of genomic DNA from the proband, proband’s sister, and proband’s mother. The boundary between exon23/intron23 is depicted. The exon sequence is in capital letters. (**D**) Alignment of the PHKA2 exon23/intron23 junction of the novel variant with the human wild type and multiple animal species. The conserved sequence in the intron 23 donor site is boxed. The mutation is in red. An alternative splicing site located 29 bp upstream, predicted in silico, is underlined in green. The putative in-frame STOP codon (taa) in intron 23 is underlined. The exon sequences are in capital letters.

Glycogen storage disease (GSD) is a rare metabolic disorder affecting glycogen synthesis or glycogen breakdown. There are more than 10 different types and common symptoms include the following: failure to thrive, hypoglycemia, and hepatomegaly. GSD types VI and IX are caused by a malfunction of the glycogen phosphorylase cascade (Figure 1A). They account for 25–30% of all GSDs [2]. GSD type VI (GSD-VI) (Hers-disease; OMIM#: 232700) is caused by mutations in the hepatic glycogen phosphorylase gene (PYGL) located on chromosome 14. GSD type IX (GSD-IX) is caused by a deficiency in phosphorylase kinase (PhK) activity, which is responsible for the activation of PYGL by phosphorylation of Ser-15 [3]. The hepatic PhK enzyme is a homotetramer, in which each monomer is itself a tetramer composed of a catalytic subunit γ and three regulatory subunits α, β, and δ. The hepatic isoform of the α subunit is encoded by the PHKA2 gene. RNA splicing of the PHKA2 in hepatocytes results in a 3708 nucleotide ORF containing 33 exons encoding a protein of 1235 amino acids. Deleterious mutations in the PHKA2 gene cause the GSD-IXa variant (X-linked inheritance; OMIM #: 306000). Reported mutations commonly include missense (43%), nonsense (17%), and in-frame deletion or insertion in various exons (37%) [4]. Intronic mutations affecting PHKA2 splicing are rare (3% in [4]); this could be partially due to the difficulties in investigating RNA splicing since it requires access to the mRNA of the affected organ (in this case, the liver). GSD-VI and IX present common symptoms and share a similar clinical management [2]; however, only GSD-IXa is an X-linked disease. An enzyme activity measurement in erythrocytes is available for GSD-IXa1, GSD-IXb, and GSD-IXc, but normal enzymatic activity does not rule out the diagnosis of GSD-IX. Invasive liver biopsy is an important diagnostic method for the confirmation of the diagnosis by enzyme activity measurements. Hypoglycemia, short stature, hepatomegaly, and increased glycogen content in the liver are typical symptoms in both diseases. The available treatment is a dietary intervention that prevents carbohydrate storage and minimizes ketosis [5].

As an aid to better diagnose and understand the disease, in this case, we applied a procedure that enables the generation of immortal fibroblasts capable of reprogramming into hepatocyte-like cells by the addition of doxycycline to cell media under standard 2D cell cultures [6]. We have implemented this methodology in fibroblasts isolated from a patient suffering from a GSD. Genome sequencing using a GSD panel revealed a G > T mutation in intron 23 of PHKA2 (c.2597 + 5G > T), potentially resulting in aberrant splicing. Hepatocyte-like cells obtained from the proband overaccumulated glycogen. We isolated the total mRNA from the reprogrammed cells and the PHKA2 mRNA sequence confirmed the insertion of 27 bp from intron 23 into exon 23, thus, resulting in an immediate STOP codon (p.Pro867*), precluding translation beyond exon 23. In parallel, we could not detect neither PHKA2 expression nor PYGL phosphorylation.

This study represents a “proof of concept” for using proband-derived hepatocyte-like cells to establish an accurate genotype–phenotype correlation and assist in the precise diagnosis of hepatic inherited diseases. In addition, this strategy allows for the derivation of a large number of diseased hepatocyte-like cells, making them amenable for use in high-throughput platforms for drug repurposing screening and toxicology.

## 2. Materials and Methods

### 2.1. Plasmids and Lentivirus Generation

The lentiviral expression vectors pLV-hTERT-IRES-hygro [7] and FUW-M2rtTA [8] were obtained from Addgene (#85140 and #20342, respectively). The lentiviral vector TetO-HHFG was described in detail previously [9]. Lentiviruses were generated in 293T cells by co-transfection of a shuttle vector with pPAX2 and pMD2.G in a 10:7.5:5 ratio. The lentiviruses were collected and concentrated using the Lenti-X concentrator, following the manufacturer´s instructions (Clontech).

### 2.2. Cell Culture and Imaging

Diseased human dermal fibroblasts (HDFs) were isolated from a punch skin biopsy from a male GSD patient. Informed consent in writing was obtained and the study protocol conformed to the ethical guidelines of the 1975 Declaration of Helsinki as reflected in the approval by the institutional ethical committee (Number 2014-0672). The HDFs were isolated, as described in detail in https://crem.bu.edu/cores-protocols/, accessed on 1 July 2022. Briefly, the skin biopsy was incubated at 37 °C in 1 mL of digestion media (DMEM-high glucose containing 20% fetal bovine serum, 23,500 U of collagenase type I, 20 mg DNAse I, and 1% penicillin–streptomycin). The next day, the digested skin was vortexed for 20 s, centrifuged at 1500× *g* for 3 min, and resuspended in 4 mL of incubation media (DMEM-high glucose containing 20% fetal bovine serum and 1% penicillin–streptomycin). The cells were seeded in a T25 flask and left untouched for 3 days.

The HDFs were immortalized by expression of the human telomerase reverse transcriptase (hTERT; HDF-T). For this purpose, the HDFs were infected with the lentivirus vector pLV-hTERT-IRES-hygro. Forty-eight hours later, 400 μg/mL of hygromycin was included in fibroblast media (DMEM containing 10% FCS). The surviving cells were expanded in 400 μg/mL of hygromycin and stocked.

To obtain the iHDF-T, the HDF-Ts were infected with a 1:1 mixture of lentivirus generated with TetO-HHFG and FUW-M2rtTA. The fraction of double-infected cells was ≈40%, as determined in a separate well by GFP-positivity after a 24-h pulse with 1 µg/mL of doxycycline (DOX). Doxycycline (Sigma-Aldrich D9891) was prepared at 1mg/mL in water and protected from light in opaque tubes. The infected HDF-Ts were then cloned by dilution cloning, as described [10]. Briefly, the cells were trypsinized and resuspended in DMEM-high glucose containing 20% fetal bovine serum and 1% penicillin–streptomycin. A suspension of 3 cells per ml was obtained by a 1/100 serial dilution. A 200-microliter cell suspension per well was seeded in a 96-well plate (0.5–1 cell/well). The plates were kept at 37 °C and 5% CO_2_ for 2 weeks with a media change after 1 week. The wells containing the cells were expanded and the DOX-inducible GFP expression in all cells was assessed by flow cytometry. One vial of the selected cell clones was thawed and the GFP-positivity was re-confirmed. Wild-type hTERT-immortalized fibroblast cells inducible to hepatocyte-like cells by a doxycycline addition (diHLC-T) were generated previously [6].

Human liver samples were obtained from three donors. Informed consent in writing was obtained and the study protocol conformed to the ethical guidelines of the 1975 Declaration of Helsinki as reflected in the approval by the institutional ethical committee (Number 2021-771-1).

### 2.3. Reprogramming into Hepatocyte-Like Cells

The diHLC-Ts were obtained by reprogramming of the iHDF-T in media containing doxycycline (DOX), as previously described [6]. Briefly, the cells were seeded at 40,000 cells/cm^2^ in collagenized plates. Twenty-four hours later, the media was switched to HMM containing 250 ng/mL of DOX and cultured for 12 days. The media was changed every other day. PYGL phosphorylation was induced by incubation with forskolin for 45 min at 50 μM.

### 2.4. Sequencing and RT-qPCR

Genomic DNA was extracted from the patient’s cultured HDFs and white cells from the mother and sister of the proband were extracted using the QIAsymphony DNA Midi Kit and QIAsymphony (QIAGEN). A genetic study of the proband was performed by next-generation sequencing using the Custom Constitutional Panel 17MB (Agilent technologies). A library preparation was carried out according to the Bravo NGS SureSelectQXT Automated Target Enrichment protocol (Agilent Technologies) for Illumina Multiplexed Sequencing. The libraries were sequenced on a NextSeq 500 System (Illumina). The obtained sequencing data were analyzed with the Alissa software tool (Agilent Technologies), which was assembled to the human genome reference GRCh37 (hg19). Variant analyses were focused on exons and donor–acceptor intron sequences of the genes involved in glycogenosis, i.e., AGL, ALDOA, ENO3, G6PC, GAA, GBE1, GYG1, GYS1, GYS2, LAMP2, LDHA, PFKM, PGAM2, PGM1, PHKA1, PHKA2, PHKB, PHKG1, PHKG2, PYGL, PYGM, SLC2A2, and SLC37A4s. The RNA was isolated from the cultured cells using the E.Z.N.A.^®^ Microelute TOTAL RNA kit (Omegabiotek). A qRT-PCR was performed, as previously described [11]. Primers were designed using the “primer-BLAST” from NCBI at https://www.ncbi.nlm.nih.gov/tools/primer-blast/index.cgi, accessed on 1 July 2022. Next, the cDNA was amplified by PCR, purified using a QIAquick PCR Purification kit (Qiagen), and sequenced by the Sanger method using forward and reverse primers (cDNA). The primer sequences are included in Supplemental information, Table S1.

### 2.5. Immunofluorescence, PAS Staining, Glycogen Content, and Immunoblotting

Immunofluorescence and PAS staining were performed as previously described [11]. Fluorescence images were taken using the Olympus FV1000 confocal mounted on an IX81 inverted microscope.

The glycogen content was determined as described elsewhere [12] and expressed as the glucose released from the glycogen. Briefly, the cells were scraped on ice into 30% KOH and then heated at 100 °C for 15 min and treated with cold ethanol to perform glycogen precipitation. Subsequently, the pellets were incubated at 37 °C for 90 min with α-amyloglycosidase (0.5 mg/mL in acetate 0.1 M pH 5) to release the glucose from the glycogen. The glucose was determined by a coupled enzymatic assay based on the action of glucose oxidase (GOD) and peroxidase (POD) as follows:

[GOD]  Glucose + H_2_O + O_2_ 🡪 gluconic acid + H_2_O_2_

[POD] H_2_O_2_ + phenol + 4-aminoantypirine 🡪 red quinoneimine dye + H_2_O

The amount of red-colored quinoneimine is measured colorimetrically at 510 nm. The results are expressed as nmol of the glycogen-derived glucose released per mg of protein.

A gel electrophoresis was run on a mini-PROTEAN cell using TGX precast gels (Bio-Rad #456-1065). The transfer was achieved in a Trans-Blot Turbo system (Bio-Rad). Immunoblotting was performed with antibodies against the PHKA2 (Sc-393491; 1/200), PYGL (Ab-198268; 1/1000), and the anti-pS15-glycogen phosphorylase (University of Dundee, 1/1000) [12].

## 3. Results

### 3.1. A Novel Mutation in the Donor Splice Site in Intron 23 of the PHKA2 Gene

The proband (II-2, Figure 1B) was referred to the hospital with a hepatomegaly at 19 months. The patient also presented elevated blood transaminases (100–180 U/L). After a liver biopsy, the patient was diagnosed as having possible GSD affecting the liver glycogenolysis (Type I, III, VI, or IX). Although the patient was asymptomatic, persistent hepatomegaly and hypertransaminasemia resulted in a new liver biopsy at the age of five. The high liver glycogen content and the undetectable phosphorylase activity were suggestive of a GSD type VI or IX. An incipient steatosis and portoportal fibrosis were also detected.

Genomic sequencing identified a hemizygotic variant in the PHKA2 gene, c.2597 + 5G > T, corresponding to the splice donor consensus sequence in intron 23. This variant had never been described in the literature (either pathogenic or not). The proband’s mother (I-1) and sister (II-1) were heterozygous carriers of the same novel variant (Figure 1C). A multiple alignment with several genomes from very distant species revealed a highly conserved donor sequence (Figure 1D), suggesting a high relevance for an accurate PHKA2 RNA splicing. In silico analysis of the WT sequence (http://www.lbgi.fr/spliceator/, accessed on 1 July 2022) predicted a donor splice site in the actual position with a 98.9% probability. A G > T substitution reduced the probability to 85%. In parallel, it also predicted an alternative donor site at position +29 bp with a 76% probability. Using the latter for the splicing would result in the incorporation of 27 bp into exon 23 with the immediate presence of a STOP codon, resulting in the truncation of the protein. In order to fully characterize the functional consequences of the mutation and the sequence of the mutated PHKA2 mRNA, we decided to derive the hepatocyte-like cells from the proband’s dermal fibroblasts.

### 3.2. Implementing a Robust and Simple Method to Generate Hepatocytes from Patient-Specific Dermal Fibroblasts

**Figure 2:**
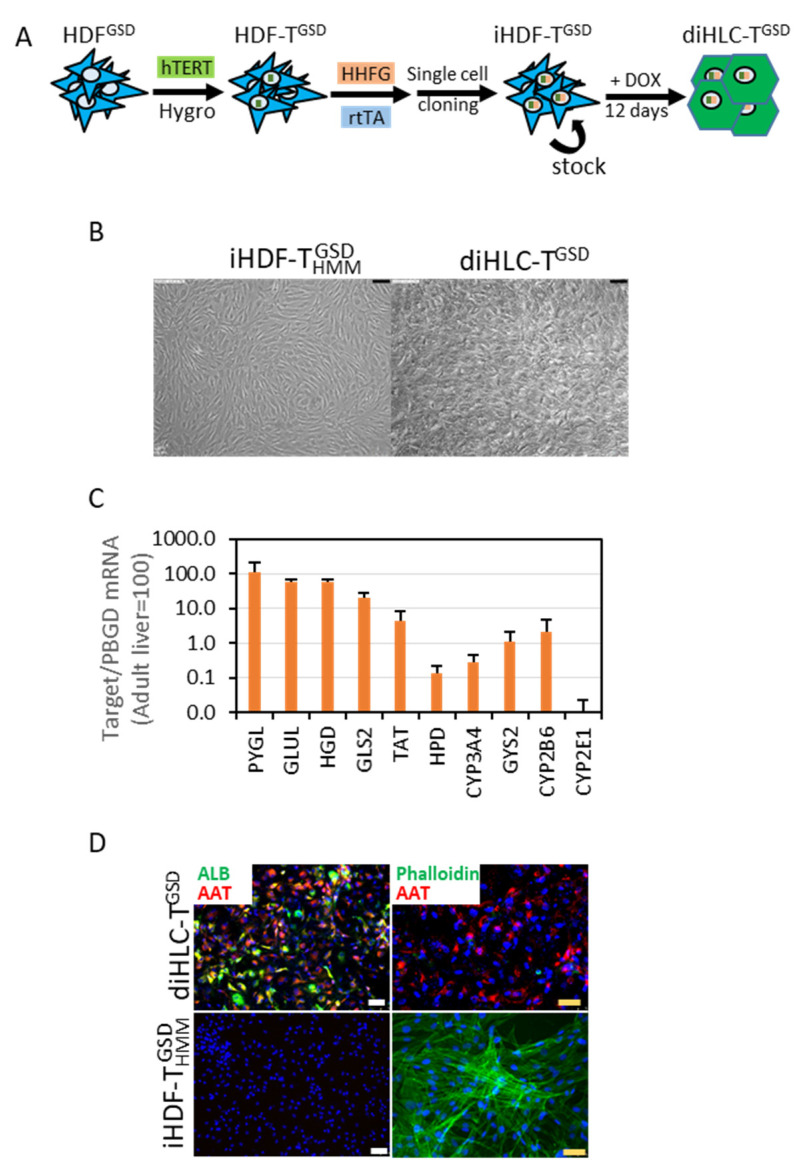
Doxycycline reprograms iHDF-T^GSD^ into hepatocyte-like cells (diHLC-T^GSD^). (**A**) A schematic representation of our reprogramming protocol. (**B**) Representative phase contrast images of iHDF-T^GSD^ cultured for 12 days without (left panel) or with (right panel) 250 ng/mL doxycycline. The black bar equals 100 μm. (**C**) mRNA level of multiple hepatic genes in diHLC-T^GSD^ quantified by qRT-PCR, normalized to PBGD, and expressed relative to the levels in the adult human liver. Values correspond to the average of three different clones (six samples each). (**D**) Representative fluorescence images of cells immunostained with antibodies against human albumin and α1-antitrypsin. Actin filaments were visualized by incubation with Alexa Fluor™ 488 Phalloidin. The nuclei were stained with DAPI. The white and yellow bars equal 75 μm and 50 μm, respectively.

We have recently published a reliable and effective procedure for the generation of an immortalized non-transformed doxycycline-inducible human fibroblast cell line capable of reprogramming into hepatocyte-like cells [6]. Human dermal fibroblasts isolated from the proband (HDF^GSD^) were first infected with a lentiviral vector constitutively expressing hTERT (Figure 2A). After a hygromycin selection, we obtained a pooled population of resistant fibroblasts expressing hTERT (HDF-T^GSD^). The HDF-T^GSD^ were then infected with a 1:1 mix of the lentiviral vectors TetO-HHFG and rtTA (reverse tetracycline receptor) and four 96-well plates were seeded at 0.5–1 cell per well comprising 240 wells (60 wells per plate). A total of seven clones grew and four of them were above 90% GFP-positive after a 48-h DOX pulse (iHDF-T^GSD^). Three of the clones were re-tested and they displayed more than 92% GFP-positivity over time.

The addition of DOX to the iHDF-T^GSD^ induced a drastic change in morphology, manifested by the loss of the typical fibroblast’s elongated morphology and the acquisition of a compact polygonal cell mass (Figure 2B). We isolated the total RNA after 10 days and analyzed the expression of 10 mRNAs as surrogate markers of the hepatocyte phenotype. These included genes encoding enzymes of tyrosine metabolism (TAT, HPD, and HGD), enzymes involved in glutamine/glutamate metabolism (GLS2 and GLUL), enzymes involved in glycogen metabolism (GYS2 and PYGL), and enzymes involved in xenobiotic metabolism (CYP2B6, CYP2E1, and CYP3A4). According to this analysis, the diHLC-T^GSD^ can be considered as fetal-like, immature hepatocytes in terms of the mRNA expression (Figure 2C). Expressions of albumin and α1-antytrypsin were detected in the diHLC-T^GSD^ by immunofluorescence (Figure 2D). A drastic reorganization of an actin filament assembly was also noted in agreement with the mentioned cell shape change.

### 3.3. diHLC-T^GSD^ Display Characteristic Features of GSD Type IX Diseased Hepatocytes

**Figure 3:**
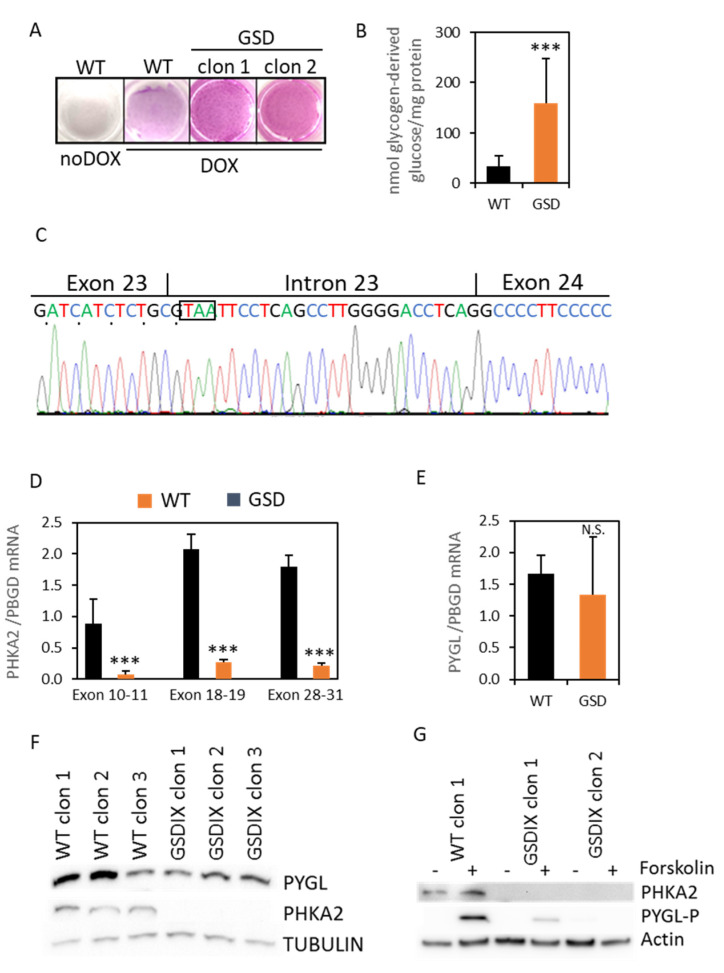
Characterization of the GSD-IX phenotype. The iHDF-T^GSD^ were reprogrammed into the diHLC-T^GSD^ by incubation in HMM media containing 250 ng/mL of DOX for 12 days. (**A**) PAS staining. (**B**) Quantification of intracellular glycogen in the mutant diHLC-T^GSD^. The glycogen content is expressed as nmol of glucose released from the glycogen digestion with α-amyloglycosidase. (**C**) Sanger sequencing of the PCR-amplified cDNA across the exon 23–24 junction showed an additional twenty-seven base pair insertion in the patient cells compared to the reference sequence. The in-frame STOP codon is boxed. (**D**,**E**) Expression of the PHKA2 mRNA, measured by RT-qPCR using primers designed in exons 10–11, 18–19, and 28–31. The expression of the *PYGL* mRNA is also shown. The values depicted correspond to the average of three different clones of reprogrammed diHLC-T and diHLC-T^GSD^. All values correspond to average plus standard deviation. (**F**) Western blot analysis of PYGL and PHKA2 in diHLC-T and diHLC-T^GSD^. (**G**) Western blot analysis of PHKA2- and Ser15-phosphorylated PYGL in diHLC-T and diHLC-T^GSD^ with and without forskolin. ***, *p* < 0.005; N.S., not significant.

The original GSD clinical diagnostics of the patient were based on the glycogen over-storage and the very limited glycogen phosphorylase activity in the liver biopsy from the patient (data not shown). To fine-tune the molecular diagnosis, we evaluated several metabolic features of the diHLC-T^GSD^. We qualitatively found an excess of glycogen deposits in the diHLC-T^GSD^ by PAS staining in comparison with the WT cells (Figure 3A). A quantitative analysis confirmed a 4–5-fold higher glycogen content (Figure 3B). Thus, the diHLC-T^GSD^ recapitulate the liver phenotype of GSD-IX.

We then focused our attention on PHKA2, since we had already found a novel SNP in the donor site splice consensus sequence in intron 23, which is potentially affecting the RNA splicing. Indeed, the mRNA sequencing revealed an inclusion of 27 bp from intron 23 into exon 23 (Figure 3C), leading to an immediate STOP codon and resulting in the truncation of the protein from exon 24 to exon 33 (p.Pro867*). Next, to get a deeper insight into the impact of the novel PHKA2 variant in the hepatocyte-like cells, we first evaluated the expression of the PHKA2 mRNA by RT-qPCR using primers designed between exons 10–11, 18–19, and 28–31. The differential expression between the WT and the mutant PHKA2 mRNA was 11-, 7.6-, and 8.2-fold, respectively (*p* < 0.05; *n* = 3; Figure 3D). Notwithstanding, the PYGL mRNA expression was not different between the WT and the diseased cells (Figure 3E). However, we could confirm the absence of the PHKA2 protein in the diHLC-T^GSD^ by Western blot in the three independently isolated clones (Figure 3F and Figure S1). The expression of PYGL was comparable to the WT clones. The PYGL switches from inactive phosphorylase B to active phosphorylase A by phosphorylation in Serine 15 by PhK. On the other hand, phosphorylation of the alpha subunits by the 3’,5’-cyclic adenosine monophosphate (cAMP)-dependent protein kinase (PKA) also relieves inhibition of the gamma subunit and, thereby, activates the enzyme [13]. To induce PhK activation in order to trigger PYGL phosphorylation in the Serine residue 15 we used forskolin, a classic activator of adenylyl cyclase that induces intracellular accumulation of cAMP. The forskolin treatment triggered the phosphorylation of PYGL in the WT diHLC-T, but it was very mild or absent in the diHLC-T^GSD^ (Figure 3G). Thus, we conclude that the patient suffers a GSD type IXa, caused by a missense mutation in the PHKA2 intron 23 splice donor site consensus sequence.

## 4. Discussion

Massive sequencing has significantly improved genetic diagnostics and allowed the identification of multiple genomic variants, some of which may have potential deleterious consequences. The clinical relevance of each variant and the final assessment by a genotype–phenotype correlation is frequently limited due to the accessibility to the affected organ. It is important to keep in mind that clinical handling and therapeutic approach can be established for multiple IMD types based only on clinical data (such as in some GSD), however, the genetic diagnosis and counseling must be based on the specific mutation, for instance, in autosomal vs. X-linked diseases (GSD type VI vs. IXa). To assist in this definitive diagnosis, where accessibility to the tissue is not straightforward, disease modeling based on cell reprogramming is a promising technology to overcome this limitation and permit an accurate and individualized diagnosis. In parallel, it allows for the development of cellular models for drug repositioning screening for IMDs.

Next-generation sequencing (NGS) has become a preeminent tool for molecular diagnosis. Whole-exome sequencing is especially useful for complex disorders with less-specific clinical findings [14]. However, in diseases such as GSDs, where the biochemical or clinical features can easily focus the diagnostic to specific pathways or genes, NGS-based target gene analysis, such as the one employed in this study, has been proven to be efficient and cost-effective [15,16,17,18]. Current molecular diagnosis based on stepwise Sanger sequencing is expensive and time-consuming, as well as it delays even more the initial diagnosis. This is mainly due to the inherent low yield of Sanger sequencing, where molecular diagnosis cannot be established for many persons in whom GSD may be suspected.

Splicing is a critical gene expression step, whereby introns must be removed from the precursor mRNA. This process is strictly controlled by multiple factors, including a variety of cis-regulatory elements, such as the 5′-donor and 3′-acceptor splice sites. Mutations in these sequences are potential pathogenic variants that can disrupt splicing and consequently lead to disease. Despite the implementation of novel algorithms and deep-learning methods, predicting the effect of the variants, even for canonical splice sites, remains limited [19]. Therefore, RT-PCR of patient RNA samples is the most direct method to test for splicing. The splice donor site consensus sequence is GT(A/G)AG. Intronic mutations of the type “+5G > T”, such as the one described here, have been reported for various diseases [19,20,21,22,23] and have always been associated with aberrant splicing. 

We have identified a novel SNP (c. 2597 + 5G > T) in the PHKA2 gene of a patient presenting hepatomegalia, hypertransaminasemia, excessive liver glycogen content, and unmeasurable liver phosphorylase activity. Intronic mutations in the PHKA2, such as the one described in this report, are rare in patients with a GSD type IX (1 in 30 reported in [4]) and not representative of the disease. We generated hepatocyte-like cells by direct reprogramming of dermal fibroblasts isolated from the proband and found aberrant splicing together with a 7–11-fold downregulation of the mRNA. The aberrantly spliced mRNA generates an ORF of 2601 bp encoding a truncated protein comprising aa1-aa866 of the WT PHKA2 (aa1-aa1235). Using an antibody raised against aa 608–778 of the human PHKA2 (www.scbt.com, accessed on 1 July 2022), we detected the full length PHKA2 in the WT hepatocyte-like cells, but not in the diHLC-T^GSD^. Concomitantly, phosphorylation of the PYGL induced by forskolin was abrogated. The C-terminal region of PHKA2 (aa1066–aa1235) share similarities with calcineurin B-like proteins [24], contributing to the regulation by Ca^2+^. Cross-linking experiments have shown that the C-terminal region and residues 724 and 981 of the PHKA2 interact with the α-subunit [25,26]. Still, we cannot rule out that the low amount of PHKA2 due to mRNA downregulation is the actual cause for the PYGL phosphorylation deficiency.

The liver, with its multiple functions, is one of the most important organs. It plays an active role in metabolism as it secretes bile that breaks down fats in the small intestine during digestion, stores and releases glucose, and synthesizes different types of proteins. In addition, the liver converts harmful ammonia into urea, processes hemoglobin, clears bilirubin, fights infections, and detoxifies medicines and other toxic chemicals [27]. Conversely to the use of more classical animal (mouse) models to study hepatic diseases, obtaining hepatocyte-like cells by direct reprogramming of patients’ somatic cells enables the generation of hepatocytes that retain every subtle feature of a given patient. Thus, studying the potential beneficial effects of novel drugs on these cells allows for a better approach to a successful personalized use of drugs.

Strategically, immortalization of HDF was achieved by expression of the human telomerase reverse transcriptase (hTERT). Telomerase shortening is the molecular clock that triggers senescence; thus, hTERT expression maintains normal HDF in a youthful state [28]. The expression of hTERT by a bicistronic IRES cassette co-expressing a hygromycin resistance gene allows for periodic iHDF re-selection with hygromycin (400 μg/mL) during amplification and stocking. Regarding the hepatic phenotype of the reprogrammed hepatocyte-like cells, RT-qPCR analyses, in comparison with our previous studies [6,9]. allow us to classify them as hybrid immature/mature hepatocytes. Importantly, glycogen metabolism is functional. We have chosen a strategy based on a DOX-inducible vector, as opposed to the individual expression of HNF4A, HNF1A, and FOXA3. Independent expression is viable, faster, and reaches similar hepatic phenotypes [29]. However, independent expression leads to elderly cell cultures, thus, co-infection must be repeated for each experiment, resulting in less reproducibility. Therefore, inducible reprogramming strategies are preferable based on their low variability and adaptation to high-throughput platforms.

Opposite to the introduction of a loss-of-function mutation in healthy (wild-type) cells, derivation from patient’s cells allows for individualized diagnostics and analyses of the subtle variances between the different gene mutations acting on a specific genetic background. It is also conceivable that this could help in evaluating personalized drug treatments prior to clinical administration. Moreover, complex multigenic disorders, such as liver steatosis [30] or lipid metabolic defects [31], where epistatic interactions or even specific combinations of single nucleotide polymorphisms play a crucial role in the disease, cannot always be obtained by targeting a specific gene [32].

Certainly, the strategy envisaged in this paper facilitates the access to the otherwise hardly accessible patient’s hepatocytes (because of ethical and biomedical restrictions) to have them in a sufficient amount to perform a more precise molecular diagnosis of the disease and to identify possible therapeutic targets, as suggested in this paper. Reprogrammed cells retain all genetic features of the disease and of the patient, which is also a great advantage to explore personalized medical treatments. This way, more realistic, close-to-patient, experimental disease models can be generated. This is a more simple and affordable strategy than attempting to generate animal models for each phenotypic variant of the disease. Thus, this way of generating a collection of cells from patients’ phenotypic variants of the disease opens a vast scenario of possibilities to improve molecular and functional diagnosis of diseased patients via minimally invasive techniques and to explore the use of drugs in a context of personalized medicine.

## Data Availability

Supporting data from this study are available through contact with the corresponding author.

## Supplementary Materials

The following supporting information can be downloaded at: https://www.mdpi.com/article/10.3390/jpm12071111/s1, Figure S1: An overexposed Western blot of PHKA2 is shown in Figure 3F; Table S1: Primers used in this study.
