# Supplementary material for: Modeling a Novel Variant of Glycogenosis IXa Using a Clonal Inducible Reprogramming System to Generate “Diseased” Hepatocytes for Accurate Diagnosis
Source: J Pers Med. 2022 Jul 7;12(7):1111. doi: 10.3390/jpm12071111 (PMC9322025; doi:10.3390/jpm12071111)
Supplement: Supplementary file 1 [file jpm-12-01111-s001.zip › gix SUPP.pdf]

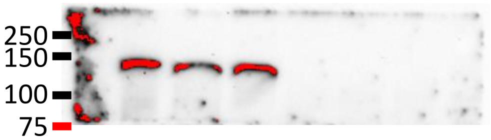

Figure S1

Figure S1. Over-exposed western blot of PHKA2 shown in Figure 3F. Red areas correspond to overexposed domains as automatically assessed by the acquiring software.
